# Impact of nanoemulsion of Ajwain-cardamom essential oils on Mortadella sausage quality during chilling (4°C) storage

**DOI:** 10.1016/j.heliyon.2025.e41643

**Published:** 2025-01-02

**Authors:** Elmira Taherzadeh, Akram Arianfar, Elham Mahdian, Sharareh Mohseni

**Affiliations:** a Department of Food Science and Technology, Quchan Branch, Islamic Azad University, Quchan, Iran; b Department of Chemistry, Quchan Branch, Islamic Azad University, Quchan, Iran

**Keywords:** Double emulsion, Preservation, Shelf-life, Texture, Sensory properties

## Abstract

Essential oils application as natural preservatives is challenging owning to low solubility and stability to harsh conditions, while incorporation of essential oils into nanoemulsion systems can effectively improve these issues. Therefore, the nanoemulsion of *ajwain* (*C. copticum*) and cardamom essential oils were fabricated through self-emulsification technique and evaluated their size, ζ-potential, antioxidative and antibacterial activities. The effect of double nanomulsion on the textural and sensorial properties of Mortadella sausage was also examined under chilling temperature (4 °C). Our goal was to improve the chilling storage of Mortadella sausage by using *ajwain* and cardamom nanoemulsion as natural preservative. By increasing the *ajwain* essential oil in the nanoemulsion, the protein and moisture of sausage increased, while the fat content decreased (17 %). Furthermore, nanoemulsion of *ajwain and* cardamom essential oils showed particle size less than 100 nm and PDI<0.5 revealing the stability of nanoemulsions. Moreover, double nanoemulsions exhibited higher antibacterial activity against *S. aureus* and IC_50_ DPPH value (107 ppm). The nanoemulsion had a greater effect on the textural properties of Mortadella, reduction in hardness (∼5300 g), and chewiness (∼2500 g mm). *Ajwain/*cardamom nanoemulsion also increased the sensory properties, particularly taste and acceptance of the Mortadella. Consequently, *Ajwain/*cardamom nanoemulsion not only improve the storage of mortadella sausage at chilling temperature due to their antioxidant and antimicrobial properties, but also has a positive effect on the red color and textural properties created a special herbal aroma, taste and odor in the Mortadella samples, which ultimately contributed to the customer-friendly product. The appropriate dose of these nanoemulsion can develop meat products at lowest amount of nitrite in Mortadella sausage formulations, although, further research should be conducted on the mechanism of action AEO/CEO nanoemulsion concerning appearance and nitrite reduction in the meat products.

## 1 Introduction

Ajwain *(Carum copticum* L.) is an annual herbaceous plant with white flowers and small brown seeds that belongs to the Apiaceae family. It is distributed in arid and semiarid regions of India, Iran, Iraq, and Egypt [1,2]. Ajwain seeds encompass an essential oil (EO) comprising about 50 % thymol, which has strong antibacterial and fungicidal effects. Furthermore, ajwain essential oil (AEO) exhibits anti-cholinergic, antioxidant, analgesic, and antihistamine properties, and it has been used to treat gastrointestinal disorders such as cramps, reflux, abdominal pain, and tumors [3,4]. Moreover, several researches have been conducted to identify the chemical composition of ajwain essential oil [[3], [5], [6], [7]]. Due to its health effects and strong antibacterial and fungicidal properties [1,8], EO of Ajwain (*C. copticum*) has found diverse applications as a seasoning and preservative agent in the food industry. These applications include the cold storage of rainbow trout and carp [[9], [10], [11], [12]], yoghurt and fermented dairy products [13], and packaging films [14].

Cardamom (*Amomum sublatum*) is a medical plant belonging to the Zibgiberaceae family, known for its pleasant aroma, flavor, and preservative effects [15]. Cardamom seeds and their essential oils are mainly used in ayurvedic medicine to treat conditions of the head, mouth and rectum as well as being used as a seasoning in foods such as coffee or tea [16,17].Cardamom essential oil (CEO) mainly consists of monoterpene compounds such as 1,8-cineol, α-terpineol, α-pinen, linalool and ester constituents α-terpinyl acetate [18,19]. It has been found that CEO has therapeutic benefits in controlling cardiovascular, pulmonary, kidney, gastrointestinal and lung disorders [20]. The therapeutic properties of CEO, along with its antifungal, bactericidal effects, as well as its pungent, warm and aromatic flavor have expanded its applications in various food products, including biofilm packaging [21] and extending shelf-life [[22], [23], [24]].

There is an increasing demand for natural preservatives instead of synthetic food additives worldwide, and industrial consultants are continuing seeking new ingredients [25]. However, due to the insoluble nature and low stability of plant essential oils like AEO and CEO against temperature, oxygen, and light, further protection methods such as encapsulation [26], complex coacervation and nanoemulsion are required [27,[28], [29]]. The use of nanoparticles of essential oils has improved solubility, stability, sensory desirability, and enhanced antimicrobial activity in the nanoemulsion systems. There is a considerable research interest in applying nanoemulsion systems due to their simple operation conditions, such as mixing, shearing, and homogenization [30,31]. However, there is limited research works on the nanoemulsion of these preservatives in food systems [15,32]. Jafarizadeh-Malmiri and coworkers have been developed a three-component ginger-cinnamon-cardamom essential oil nanoemulsion as a natural food preservative [27].

To the best of our knowledge, there is no research work on the double nanoemulsion of AEO and CEO in the food systems. The aim of the current work is to prepare a double nanoemulsion of ajwain essential oil (AEO) and cardamom essential oil (CEO) to create a natural food preservative for mortadella sausage as a model system, addressing the significant knowledge gap regarding the combined efficacy and application of AEO and CEO in nanoemulsion form, particularly in enhancing the quality, safety, and shelf life of meat products while overcoming the stability and solubility challenges traditionally associated with plant essential oils. Therefore, antioxidative and antibacterial properties of the essential oil nanoemulsion were analyzed to get information on its application in mortadella sausage.

## 2 Materials and methods

### 2.1 Materials

Ajwain seeds were collected from wild plants in Uremia Mountains (Iran) in mid-July 2018. Permission for collecting the Ajwain seeds was obtained from Research Institute of Forests and Rangelands (TARI) and a voucher specimen was also submitted to the herbarium of the Research Institute of Forests and Rangelands (TARI), Tehran, Iran. The experimental protocol was approved by Research Institute of Forests and Rangelands (TARI), Tehran, Iran and formal identification of the Ajwain was performed by Jalali [33]. Cardamom seeds and sunflower oil were prepared form national market Nina TM (Mashhad, Iran). Tween 80 (HLB 15), glycerol (HLB 4.5), 2,2 diphenyl-1-picrylhydrazyl (DPPH), and all other reagents for GC-Mass Spectroscopy were analytical grade and purchased from Merck (Merck KgaA, Darmstadt, Germany). Microbial media cultures were provided by Liofilchem (Roseto degli Abruzzi, Italy).

### 2.2 Essential oil extraction

Ajwain and cardamom seeds were completely dried and powdered to particle size of about 500 μm in order to increase the mass transfer rate for essential oil extraction. Then, 30 g of dried powder along with 700 ml of distilled water was hydro distillated using a Clevenger-type apparatus for 4 h, according to the previous work [11]. The volatile was collected over anhydrous sodium sulphate to improve the recovery and stored at 4 °C. The yield of the essential oil of Ajwain on dry basis was approximately 3 % (w/v) which was similar to the previous work [14].

### 2.3 GC-MS analysis

The chemical compositions of AEO and CEO were analyzed through gas chromatography-mass spectrometry (GC-MS). The GC-MS analysis was conducted using a gas chromatograph (7890B, Agilent, Santa Clara, CA, USA) coupled with a mass detector (5977A, Agilent technologies, USA). The gas chromatograph was outfitted with a HP 5-ms capillary column (phenyl methyl siloxane, 30 m length, 0.25 mm inner diameter, and 0.25 μm layer thickness, Agilent technologies). The injector temperature was set at 270 °C, and the oven temperature was programmed to increase from 60 (0 min) to 200 °C at a rate of 5 °C/min. Helium served as the carrier gas at a flow rate of 1 mL/min, with an injection volume of 1 μL. The mass spectrometer operated in ionization mode at 70 eV, with the interface temperature at 280 °C, and the mass range scanned between 35 and 500 *m*/*z*. The essential oil components were identified by comparing their retention indices (C_7_ to C_20_ n-alkanes) and mass spectral fragmentation patterns, which were used to calculate the Kovats indices from the gas chromatographic analysis [[10], [34]].

### 2.4 Preparation of double nanoemulsions of AEO and CEO

According to our preliminary work [35], the AEO and CEO nanoemulsions were prepared through the self-emulsification technique. Firstly, Tween 80 (15 %) and glycerol (6 %) were mixed completely through stirring over a magnet stirrer (IKA Plate, RCT digital, Germany) for 5 min at a speed of 500 rpm. The AEO and CEO were then added in various ratios 0:100, 30:70, 40:60, 50:50, 60:40, 70:30, and 100:0 (Table 1) to the Tween 80 and glycerol mixture to acquire total concentration of 1 % w/w and stirred for 15 min. This mixture was subsequently inserted dropwisely to the deionized water (78 %) which was located in a water bath. The mixture was placed over a magnet stirrer and let to completely mix until a homogenous translucent solution was achieved and kept it at room temperature to equilibrate and became a transparent appearance [36].

**Table 1:** Experimental design, particle size, poly dispersibility index (PDI), ζ-potential, anoxidative and antimicrobial properties of ajwain essential oil (AEO) and cardamom essential oil (CEO) nanoemulsions.^a^

sample code	AEO (%v/v)	CEO (%v/v)	Size (nm)	PDI	Zeta potential (−)	IC_50_ DPPH (ppm)	Antibacterial activity	
							S. aureus (mm)	E.coli (mm)
1	0	100	98.05 ± 0.03e	0.33 ± 0.12c	−8.62 ± 0.17a	38.72 ± 0.95f	32.5 ± 0.6c	12.5 ± 0.8d
2	30	70	75.08 ± 0.03c	0.15 ± 0.08b	−6.03 ± 1.17b	53.43 ± 1.01e	37.3 ± 0.4c	13.3 ± 0.4d
3	40	60	98.47 ± 0.07b	0.37 ± 0.22b	−5.54 ± 0.65c	87.25 ± 1.92c	44.0 ± 0.7b	20.8 ± 0.5b
4	50	50	99.02 ± 0.04a	0.36 ± 0.06a	−6.67 ± 0.23b	107.54 ± 1.33b	65.3 ± 0.9a	25.6 ± 0.3a
5	60	40	77.35 ± 0.17c	0.34 ± 0.05a	−7.87 ± 0.33a	132.87 ± 1.73a	74.1 ± 0.2a	24.1 ± 0.1a
6	70	30	67.55 ± 0.23d	0.43 ± 0.15a	−3.83 ± 0.45d	124.94 ± 1.03a	55. 2 ± 0.5b	17.4 ± 0.2c
7	100	0	98.89 ± 0.13d	0.37 ± 0.17b	−3.43 ± 0.14d	47.65 ± 0.88d	47.8 ± 0.7b	17.3 ± 0.5c

IC_50_ DPPH shows the concentration of active compound required for inhibition of 50 % of the free radicals.

a Alphabetic small letters in each column show significant differences in 95 % confidence. The values in the tables are provided in mean ± SD.

### 2.5 Turbidity analysis

Turbidity measurements of the nanoemulsions were performed using a UV–visible spectrometer according to Jamshidian and Rafe [28]. The absorbance of the samples was measured at 600 nm at room temperature and distilled water was used as a blank solution.

### 2.6 Particle size, polydispersity index and ξ-potential analysis

The hydrodynamic diameter and ξ-potential of the nanoemulsions were assessed using a Malvern dynamic light scattering (DLS) (Nano-ZS, Malvern Instruments, Malvern, Worcestershire, UK). Three replicates were carried out for each microcapsule at 25 °C, and the mean values were reported as z-diameter (Anarjan, 2018).

### 2.7 In vitro antimicrobial assay

The antimicrobial efficacy of AEO and CEO against 2 pathogenic bacteria including *Escherichia coli* O157:H7 PTCC 1860 and *Staphylococcus Aureus* PTCC 1917 was analyzed using agar disk diffusion assay. The bacterial suspension was overnight in Muller-Hinton broth which was adjusted to 0.5 McFarland turbidity standard (10^8^ CFU/ml). It was then spread on the surface of Muller-Hinton agar plate. The sterile blank disks were soaked in 20 μL of nanoemulsions of AEO and CEO (1 and 2 %), sterile water as negative control, and streptomycin (100 mg/mL) as positive control, dried for 10 min and located on inoculated plates. The plates were subsequently incubated overnight at 35 °C. The microbial inhibition was evaluated by the inhibition zone diameter around the disk [[12], [37], [38], [39]].

### 2.8 Scavenging activity

The antioxidant activity of nanoemulsions of AEO and CEO was assessed using DPPH according to the Rinaldi et al. [40]. In brief, 1.0 mL of each nanoemulsion was mixed with methanolic solution of DPPH (0.004 %). The mixture was kept at room temperature and dark condition for half an hour. The absorbance of the mixture was measured at 517 nm and compared with pure methanol and the scavenging activity was determined using the following equation (Eq. (1)):

(1) Scavenging activity (%) = (A_c_-A_s_/A_c_)∗100

Where A_c_ and A_s_ are the absorbance of methanolic DPPH solution and sample, respectively.

### 2.9 Mortadella sausage preparation

Mortadella sausage was prepared according to the previous work [35]. Briefly, beef was ground (model PJ22, Jamar Ltd. Brazil) in a plate with 3 mm openings. The mortadella sausage was prepared as follows: beef (60 %), garlic powder (1 %), sodium chloride (1.6 %), sodium tripolyphosphate (STPP) (0.035 %), sodium nitrite (0.01 %) along with ice/water mixture (16 %) were placed in the cutter (model KJ20, Jamar, Brazil) to extract the myofibril proteins at higher speed (3 min). Then, sodium lactate (1 %) and pasteurized albumin liquid egg (4 %) were added to the mixture, and the oil (8 %) was slowly added to the mixture and the process continued for about 120 s to form a uniform emulsion. The remaining additives including spices (1 %), sugar (1 %), wheat flour (2 %), starch (4 %) and ascorbic acid (0.04 %) were added slowly followed by comminution at the speed of 1500 to 3000 rpm for 2 min. The nanoemulsions of AEO and CEO at levels of 1, 2 and 3 % were also mixed to this formula. In order to obtain a uniform emulsion, the comminution process was carried out for 6 min. However, the temperature was controlled at 0 °C at the beginning of the process; the meat's emulsion temperature did not passed 12 °C during homogenization. Then, meat dough was stuffed into water proof polyamide casings (φ = 60 mm) using a stuffing machine (model PJI-09, Jamar Ltda, Brazil). The mortadella sausages were then placed in cooking chamber until the internal temperature reached 72–74 °C. The sausages were then cooled to 4 °C in an ice bath. After 12 h, the samples were sliced and vacuum-packed in polyethylene liners. Finally, all samples were stored at 4 °C until further experiments. The sausages were stored at 4 °C and microbial, physicochemical, sensorial and textural properties were periodically examined at 1 and 30 days of storage. Duplicate samples were analyzed per each replicate.

#### 2.9.1 Physicochemical properties and proximate composition of sausage

The chemical composition of Mortadella sausage, including moisture, protein, fat, and ash content, was determined according to the Association of Official Analytical Chemists [41]. The moisture content was determined using a convection oven at a temperature of 105 °C (AOAC Method 950.46). Nitrogen content was measured by digestion with Micro-Kjeldal, and protein was calculated by multiplying N × 6.25 (AOAC Method 920.152). Fat content was measured by the Soxhelt method (AOAC Method 963.15), and mineral content was determined by combustion of the samples in a muffle furnace at 550 °C (AOAC Method 940.26). The nitrite content of the samples was determined by spectrophotometry (UV-1800, Shimadzu, Hitachi, Japan) (AOAC Method 902.167). The pH of the samples was determined during storage using a pH meter (Inolab, Weilheim, Germany). All experiments were performed in triplicate.

#### 2.9.2 Shelf life evaluation by microbial analysis

Microbial experiments are included as total viable count (TVC), *E.coli*, and lactic acid bacteria, which were done for all samples three times during the storage period (1, 7, 14, 21, 28, and 35 days after production). So, sausage samples (10 g) were transferred aseptically into sterile bags to homogenize with 90 mL of Ringer's solution (0.85 % NaCl) and stomached (Interscience Ltd., France) for 2 min s. To obtain a microbial count, other decimal dilutions were made with Ringer's solution, and 0.1 mL of the appropriate dilutions was transferred onto plates. Incubation at 37 °C for 36 h was used to obtain the total viable counts (TVC), *E.coli*, and lactic acid bacteria. TVC in PCA medium, *E. coli* EMBA medium, and lactic acid bacteria in MRS medium were prepared according to the ISIRI (Iraian standard organization, 2303). Microbial enumeration data were expressed as log_10_ cfu/g in an acceptable range of 30–300 colony units per plate by a microcounter device [[42], [43]].

#### 2.9.3 Textural properties

Texture profile analysis (TPA) of Mortadella sausages was performed using a texture analyzer (model TA-TX2, Stable Micro Systems, Surrey, UK.) equipped with a 5 kg load cell as described by previous works with slight modifications [[44], [45], [46]]. Samples were cut into cylinders 1.5 cm high and 2.5 cm wide. The calibration settings were 5 kg load cell with return trigger path at 1.5 cm. The measurement mode settings of compression (pretest; test and posttest) were set to a speed of 1.0 mm/s; strain at 50 %, trigger type at auto 10 g, time intervals between cycles 2 s, and cylinder probe 25 mm. The cylinders of sausages with dimensions of 15 mm height and 25 mm width were positioned straight. From the force-time curves of TPA, the parameters including, hardness, cohesiveness, springiness and chewiness were measured [46].

#### 2.9.4 Sensorial evaluation

A multi-point hedonic sensory test was used to evaluate the sensory qualities of the Mortadella sausages [12]. Briefly, the sensory evaluation of the sample was performed on the 7th day of storage at 4 °C. Mortadella sausages were sliced approximately 3 mm thick and served separately to each panelist under white illumination. Samples were served to consumers on white ceramic plates accompanied by water and crackers to cleanse the palate. The informed consent form must be signed by each participant per the instructions. A panel of 30 untrained judges with expertise in the meat industry was used to evaluate the products. Consumer acceptability of the samples was assessed using a 9-point hedonic test of actual acceptability. The samples were evaluated on four attributes including color, taste, texture and overall acceptability. The experiment was evaluated in the form of a non-parametric analysis according to the continuous and exact number rule.

### 2.10 Statistical analysis

All experiments in sections 2.5 to 2.10 were performed in triplicate and data were analyzed by ANOVA using the linear model of SAS software version 9.1. Duncan's multiple range tests were used to compare differences between means, and significance was defined as P ≤ 0.05.

### 2.11 Research involving plants

Authors confirm that the study on plants in this research, including the collection of plant materials (Ajwain seeds), complies with relevant institutional, national, and international guidelines and legislation. The experimental protocol was approved by Research Institute of Forests and Rangelands (TARI), Tehran, Iran. Formal identification of the plant material was performed by Jalali [33]. Furthermore, Authors confirm that the research for sensory analysis was performed in accordance with Declaration of Helsinki, and informed consent was obtained from all participants or their legal guardians.

## 3 Results and discussion

### 3.1 Chemical analysis of essential oils

Chemical compounds of cardamom and ajwain were evaluated qualitatively on GC-MS by identifying all the volatiles. The ajwain and cardamom essential oil components along with retention time and percent are provided in supplementary data. The GC profile of the ajwain and cardamom essential oil is also given in supplementary data. The GC-MS results showed 16 components which thymol (58.30 %), ρ-cymene (21.11 %) and γ-terpinene (14.72 %) were the main constituents in AEO. These results were in agreement with previous works [10,47,48]. It has been found that thymol as the main component in ajwain was in range of 35–63 % depending on its place of cultivation [47]. However, carvacrol, γ-terpinene, and p-cymene have been also reported as the main components of Iranian ajwain oil [5].

It has been found that 1,8-cineole as oxygenated monoterpene is the highest compound in the green cardamom (55.4 %), Ethiopian cardamom (51.8 %), and black cardamom (41.7 %) [49]. The other main constituents of cardamom essential oil are monoterpene hydrocarbons (36.9 %), α-terpinyl acetate (28.6 %). These results and the chromatographic profile are in line with previous works [[49], [50]]. Several reports have shown that 1,8-cineole and α-terpinyl acetate are the main compounds in the green cardamom [[51], [52], [53]].

### 3.2 Particle size, zeta potential, antioxidative and bactericidal activities

Particle sizes, PDI, ξ-potential, antioxidative and bactericidal activities are given in Table 1. To enhance water solubility, bioactivity, cell permeability, and absorption, the essential oils of AEO and CEO were incorporated into nanoemulsion systems [54,55]. The mean particle size of AEO and CEO nanoemulsions were approximately 100 nm (Table 1) indicating that self-emulsification is an effective technique for preparation pure or binary essential oil nanoparticles. Notably, the nanoemulsions exhibited diameters ranging from 67 to 99 nm. The zeta potential was found to increase from the CEO to AEO nanoemulsions. According to Table 1, AEO and CEO nanoemulsions had a significant effect (P < 0.05) on size, PDI, ξ-potential, as well as antioxidative and bactericidal activities. PDI values, which indicate the width of the particle size distribution, were all below 0.5, demonstrating better stability of the nanoemulsions. All samples exhibited a PDI of less than 0.5, indicating that they were homogeneous and had a monomodal particle size distribution. Importantly, all samples displayed negative zeta potential values.

The antioxidant and antimicrobial activities of essential oils can be attributed to their active phenolic and flavonoid constituents. Furthermore, the antioxidant activity can be enhanced by synergistic effects among the compounds. DPPH assay is a reliable technique for determining the antioxidant activity of bioactive compounds, with results expressed as IC_50_, representing the concentration of active compound required to inhibit 50 % of free radicals. The IC_50_ of the nanoemulsions ranged from 38.7 to 132.5 mg/mL. Based on analysis of variance (AONVA) results, the double nanoemulsions showed the highest antioxidative effect (P< 0.05), suggesting significant chemical interactions between CEO and AEO. The lowest IC50 was recorded for the CEO nanoemulsion, indicating a correlation between particle size and PDI with IC50. Similar findings have been reported for single cardamom essential oil nanoemulsion, which displayed reduced antioxidant activity [[27], [43]].

Furthermore, CEO and AEO nanoemulsions demonstrated antimicrobial activity against *S. aureus* and *E. coli,* representative Gram-positive (g+) and Gram-negative (g^−^) bacteria, respectively (Table 1). The binary nanoemulsions exhibited enhanced antibacterial activity against *S. aureus.* Our results indicated that the antimicrobial effectiveness of the nanoemulsions was greater against g + strains compared to g^−^ bacteria [[27], [43]]. Among the tested pathogens, *S. aureus* was the most susceptible, followed by *E. coli*. The reduced sensitivity of g^−^ bacteria can be related due to the presence of lipopolysaccharide in their outer membrane [12]. The bactericidal effect of CEO and AEO is mostly interrelated to their phenolic compounds, which disrupt the bacterial membrane [[12], [56]].

According to the size, antioxidative, and antibacterial activities of the AEO and CEO nanoemulsions, the formulations in ratios of 60:40, 50:50, 40:60 and 100:0 were incorporated into Mortadella sausage to evaluate their potential as substitutes for nitrite.

### 3.3 Proximate composition and technological properties

Chemical characteristics of Mortadella-type sausages are summarized in Table 2. All samples were prepared with 12 % protein and 30 % fat content. Notably, increasing the concentration of CEO in the nanoemuslions led to a significant increase in both protein and moisture contents. In fact, the higher levels of CEO resulted greater moisture and protein content compared to the control group (P < 0.01). The incorporation nanoemulsions in ratios of 60:40, 50:50, and 40:60 resulted in a significant decrease in fat content, measuring 19.57 %, 18.27 % and 17.08 %, respectively. Although the CEO/AEO nanoemulsion did not significantly exert a significant effect on the mineral content of the samples, the use of beef instead of pork contributed to a reduction in fat content, consistent with findings from previous work [57]. In addition, cholesterol reduction in the Mortadella sausages was observed when animal fat was substituted with various types of vegetable oils [57]. These findings align with the previous works, such as those on trout treated samples with sunflower oil and Zataria multiflora essential oil [58], and lamb loin treated with olive oil nanoemulsion [12].

**Table 2:** Effect of nitrite substitution by ajwain essential oil (AEO) and cardamom essential oil (CEO) nanoemulsions on chemical composition, cooking loss, and emulsion stability of mortadella sausages.^a^

Parameters	AEO/CEO double nanoemulsion				p-value
	100:0	60:40	50:50	40:60	
Moisture (%)	57.66 ± 0.67d	58.72 ± 0.87c	59.74 ± 0.75b	60.64 ± 0.84a	∗∗
Fat (%)	20.28 ± 0.68a	19.57 ± 0.34b	18.27 ± 0.35c	17.08 ± 0.97d	∗∗∗
Protein (%)	13.76 ± 0.21 d	13.82 ± 0.47c	14.96 ± 0.66 b	15.27 ± 0.75a	∗∗
Ash (%)	2.11 ± 0.08c	2.08 ± 0.11b	2.15 ± 0.24c	2.17 ± 0.14a	a
pH	6.10 ± 0.02a	6.12 ± 0.01a	6.08 ± 0.03b	6.05 ± 0.05c	∗∗
Residual nitrite (ppm)	32.67 ± 2.52a	30.15 ± 1.40b	28.37 ± 1.22c	26.44 ± 1.47d	∗∗∗
Cooking loss (%)	8.43 ± 0.08a	7.12 ± 0.22b	6.56 ± 0.14c	6.42 ± 0.11d	∗∗
E.S. Fat exudation^b^	1.55 ± 0.41a	0.92 ± 0.11b	0.73 ± 0.08c	0.45 ± 0.05d	∗∗
E.S. water exudation	7.54 ± 0.21a	6.91 ± 0.31b	5.77 ± 0.24c	4.49 ± 0.12d	∗∗

P-value: ∗∗∗ (*P* < 0.001), ∗∗ (*P* < 0.01), ∗ (*P* < 0.05).

a The averages with S.E. within a parameter followed by an alphabetic significant difference by Duncan's multiple range tests (*P* > 0.05). The values in the tables are provided in mean ± SD.

b E.S.:Emulsion stability.

**Fig. 1:**
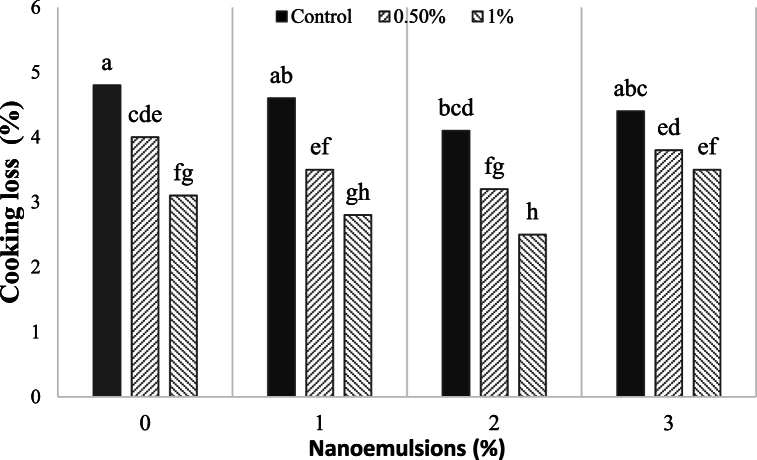
Effect of CEO/AEO nanomulsions on the cooking loss of the Mortadella-type sausage. Different letters showed the statistical significance among the treatments (*P* < 0.05).

The application of beef in the Mortadella-type sausage significantly reduced the fat content, and increasing the CEO/AEO nanoemulsion also resulted in reduced cooking (Fig. 1). The effect of AEO content in the nanoemulsion on cooking loss is illustrated in Fig. 1, which shows that cooking loss decreased with increasing AEO content. Treatments with 1, 2 and 3 % CEO/AEO nanoemulsion showed a reduction in cooking loss compared to the control, indicating improved emulsion stability. This enhancement in technological quality may be related to the collagen present in beef, which interacts with proteins to form a stable matrix that minimizes water and fat exudation from the meat. Similar improvements in the technological quality of meat processed products have been reported with soybean oil oleogel and rice bran wax used as fat replacers [59]. Similar reductions in fat content by using pork skin have also been reported [[60], [61], [62]]. It is noteworthy that as the concentration of CEO/AEO nanoemulsion increased in Mortadella-type sausages, the levels of residual nitrite decreased. While there is limited research on the reduction of nitrite by CEO/AEO nanoemulsion, previous studies indicated reductions of 3.9 %–38.8 % through gamma irradiation [63].

The effect of CEO/AEO nanoemulsion on water holding capacity (WHC) and pH of sausage is shown in Table 3. As the concentrations of AEO and CEO/AEO nanoemulsion increased, the WHC also improved due to their absorption properties. However, WHC decreased during storage due to water loss. On the first day post-assembly, the pH of the Mortadella sausage ranged from 6.10 to 6.23 (Table 3), with the samples containing 60:40 and 50:50 ratios exhibiting lower pH values compared to the control group, likely due to the lower pH of the CEO/AEO nanoemulsion. CEO/AEO nanoemulsion. Alves et al. (2016) evaluated the pH effects of replacing 80 and 100 % of the pork fat in Mortadella sausages with pork skin and green banana flour, finding that the pH increased from 6.11 to 6.39 after 30 days, which could be related to ammonium generation from increased proteolysis during storage [62]. Nevertheless, the pH levels of all treatments remained within the acceptable range for this type of product [64].

**Table 3:** Effect of ajwain essential oil (AEO)/cardamom essential oil (CEO) nanoemulsion on the water holding capacity (WHC), pH, and textural properties of mortadella during storage (4 °C, 30 days).^a^

Properties	Storage (day)	CEO/AEO nanoemulsion			
		Control	1 %	2 %	3 %
**WHC (%)**	1	88.5 ± 0.33^a^	88.3 ± 0.2^a^	88.1 ± 0.35^a^	89.1 ± 0.22a
	30	85.3 ± 0.65^abc^	84.6 ± 0.9^bcd^	85.9 ± 0.4^ab^	87.2 ± 0.3a
**pH**	1	6.2 ± 0.05^ab^	6.21 ± 0.01^ab^	6.23 ± 0.04^a^	6.2 ± 0.1ab
	30	6.08 ± 0.02^ab^	6.04 ± 0.05^abc^	6.05 ± 0.01^abc^	6.1 ± 0.03a
**Hardness (g)**	1	5255±3^a^	5190±5^bc^	5150±6^ef^	5092 ± 16g
	30	5389 ± 12^c^	5395 ± 16^c^	5350 ± 11^de^	5322±8ef
**Cohesiveness (−)**	1	0.70 ± 0.02^bc^	0.71 ± 0.00^bc^	0.75 ± 0.01^ab^	0.79 ± 0.03a
	30	0.66 ± 0.00^cd^	0.68 ± 0.00^bc^	0.70 ± 0.00^ab^	0.75 ± 0.03a
**Springiness (mm)**	1	0.81 ± 0.10^ab^	0.82 ± 0.03^ab^	0.84 ± 0.05^ab^	0.85 ± 0.01a
	30	0.75 ± 0.03^a^	0.69 ± 0.04^ab^	0.75 ± 0.01^a^	0.69 ± 0.06ab
**Chewiness (g.mm)**	1	2979.5 ± 22^d^	3021.6 ± 16^c^	3244.5±6^b^	3419.3±8a
	30	2667.6±9^c^	2531.3 ± 15^d^	2808.7 ± 11^a^	2754.2±6b

a Alphabetic small letters within a column showed the statistical significant difference (*P* < 0.05). The values in the tables are provided in mean ± SD.

### 3.4 Textural properties

Sausage texture significantly influences consumer acceptance, often being more critical than color and flavor. Textural properties can be classified into mechanical properties (hardness, springiness, cohesiveness, and chewiness), geometric properties (shape and size), and other attributes (moisture and fat content). The effects of CEO and AEO nanoemulsion on the textural properties of beef Mortadella stored at 4 °C for one month are detailed in Table 3.

Hardness is defined as the maximum force exerted during the initial compression of the sample. The addition of CEO/AEO nanoemulsion significantly increased the hardness of Mortadella on the first day of production compared to the control sample (Table 3). Storage time also had a significant impact on hardness (P < 0.05), with hardness increasing over 30 days, particularly in the control sample (from 5200 to 5516 g). This increase in hardness is attributed to factors such as starch retrogradation and enhanced hydrogen bonding, as well as product syneresis and water loss from the tissue [65]. Notably, at the end of the storage period, samples containing 40:60 and 50:50 CEO/AEO nanoemulsions exhibited less hardness than the control, likely due to improved water absorption and retention by the hydrocolloid constituents.

Springiness measures the extent to which a sample recovers its height after compression. The incorporation of CEO/AEO nanoemulsion increased springiness, particularly at a 1 % concentration. However, at 3 %, springiness decreased compared to the control due to excessive softness compromising cohesion (Table 3). Storage time had a significant effect, with springiness initially stable until declining over 30 days as texture cohesion diminished. The maximum recorded elasticity was 0.75, which was not significantly different from the control (0.50) (P > 0.05). These findings align with previous research showing that rosemary essential oil had no significant impact on turkey sausage texture [66].

Cohesiveness was significantly affected by the addition of CEO/AEO nanoemulsion (P < 0.05), enhancing the cohesiveness of the samples compared to the control. It is noteworthy that while cohesiveness was improved, storage duration did not significantly affect it (P > 0.05). On the production day, the highest cohesiveness values were noted in treatments with 1 % and 2 % nanoemulsion (0.75 and 0.79, respectively), compared to the control (0.55). Higher concentrations of the nanoemulsion fostered greater molecular interactions within the formulation, thus increasing cohesiveness. Previous studies also observed increased cohesiveness in meat products enriched with chitosan and essential oils [60,67].

Chewiness, a product of hardness, springiness, and cohesiveness, indicates the energy needed to chew solid foods. The texture profile analysis demonstrated that sausages containing 2 % nanoemulsion were chewier than the control due to increased hardness and stability. Although the CEO/AEO nanoemulsion generally enhanced chewiness, the samples at 1–3% were still chewier than the control at the end of storage. Conversely, storage duration significantly reduced chewiness (P < 0.05), paralleling changes in hardness and springiness (Table 3). Similar trends were reported in studies showing that textural properties of pork sausages deteriorated during refrigerated storage but were stabilized by chitosan [68].Overall, replacement of pork fat with beef significantly enhanced the hardness and chewiness of Mortadella sausages while having minimal effects on cohesiveness and springiness (P > 0.05). This trend aligns with previous findings, where using pork skin and vegetable oleogels in meat products yielded similar textural improvements [[60], [61], [62]].

### 3.5 Shelf-life evaluation

**Fig. 2:**
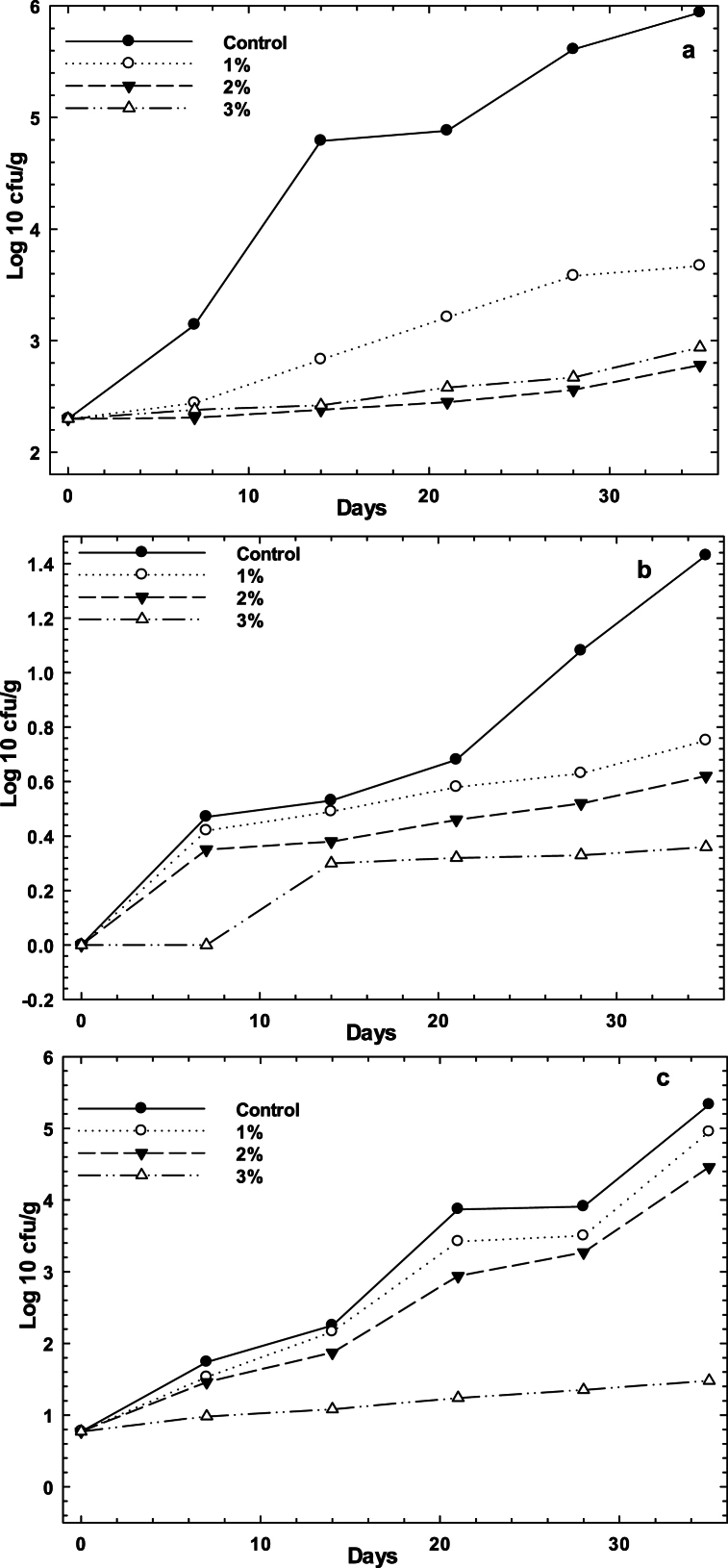
Effect of nanoemulsions of AEO/CEO (1, 2 and 3 %) on microbial counts (cfu/g) of mortadella sausage during storage at refrigerator (4 °C), Total vial count (a), *E.Coli* (b) and lactic acid bacteria (c).

Effect of CEO/AEO nanoemulsion on Mortadella sausage during storage at refrigerator (4 °C) was evaluated through surveying the microbial growth in a month (Fig. 2a–c). According to the results, it was found that there was significant reduction in total viable count (TVC) after 24 h of storage for CEO/AEO nanoemulsions (P < 0.05). The initial TVC, *E.Coli* and LAB of samples were 2.31, 0 and 0.77 log cfu/g in control samples. However there was an increasing in the TVC, *E.Coli* and lactic acid bacteria (LAB) counts during storage at refrigerator, by using the CEO/AEO nanoemulsion to the Mortadella samples, a reduction of TVC, *E.Coli* and LAB was found which are in agreement with the similar works on fish fillets [69] and carp samples [[10], [70]]. Among the CEO/AEO nanoemulsions, by increasing the amount of the CEO/AEO in the mortadella sausage, the TVC, *E.Coli* and LAB counts were reduced. These findings emphasized the antimicrobial effect of essential oils which was obtained in section 3.2 and Table 1 and confirmed the antimicrobial activities of the CEO/AEO nanoemulsions.

### *3.6* Sensory evaluation

The sensory characteristics of a sausage are one of the most important acceptance factors from the consumer's point of view. However, if the changes in the food formulations are successful in terms of the various test results, but fail in the sensory tests, they are not really applicable. The results of the sensory evaluation of Mortadella samples stored at 4 °C for 30 days are presented in Table 4. The sensory parameters of beef Mortadella were evaluated in terms of color, texture, flavor and overall acceptability using a nine-point hedonic scale. The results showed that on the first day of storage, all treatments had good sensory scores according to the panelists. The changes in sensory characteristics and their decreasing trend continued slowly until day 20, so that almost all samples were acceptable to the panelists, although there were differences between treatments. Significant changes in the sensory characteristics that led to the unacceptability of some samples occurred during the last 10 days of storage, and the effectiveness of chitosan and CEO/AEO nanoemulsion in improving the quality of sausage samples was observed during this period. For example, at the end of the storage period, the highest apparent color score of Mortadella belonged to control (6.8 ± 0.05) and (7 ± 0.3) samples and this difference was not statistically significant (P > 0.05). 1 % and 3 % with 6.5 ± 0.3 color score also had no significant difference with the control sample (P > 0.05) [71]. The results of the color sensory analysis are consistent with the instrumental analysis of the samples, as shown in Table 4, the highest values of redness (a∗) were observed in the treatments with high concentration of CEO/AEO nanoemulsion (treatments 1, 2 and 3 %), which were at the same level with the control sample (P > 0.05). One of the main effects of nitrite in meat products is the red color of cured meat products. A compound known as nitrosylmyoglobin is formed when sodium nitrite combines with the iron found in both myoglobin and metmyoglobin to produce color and the color of cured meat [[72], [73]]. Therefore, as mentioned earlier, reducing nitrite levels in meat products should be done more carefully, which means using alternatives that compensate for the apparent color reduction in addition to the preservation effect on meat products.

**Table 4:** Effect of cardamom essential oil/ajwain essential oil nanoemulsion on the color and sensorial properties of mortadella during storage (4 °C, 30 days).^a^

Properties	Storage (day)	CEO/AEO nanoemulsion			
		Control	1 %	2 %	3 %
**L**^a^	1	59.1 ± 0.3^ab^	60.8 ± 0.2^b^	62.8 ± 0.2^ab^	61.5 ± 0.4a
	30	63.4 ± 0.2^abc^	63.5 ± 0.3^ab^	64.1 ± 0.2^bcd^	63.4 ± 0.3a
**a**^a^	1	21.6 ± 0.1^a^	21.2 ± 0.1^ab^	21.5 ± 0.3^cd^	21.8 ± 0.1bc
	30	21.8 ± 0.1^a^	21.5 ± 0.1^a^	20.5 ± 0.2^a^	21.7 ± 0.3a
**b**^a^	1	15.5 ± 0.1^cd^	15.6 ± 0.1^cd^	15.6 ± 0.2^cd^	16.4 ± 0.1d
	30	15.3 ± 0.1^bcd^	16.7 ± 0.3^e^	16.6 ± 0.1^ab^	16.9 ± 0.1a
**Color**	1	7.1 ± 0.3^ab^	6.8 ± 0.2^b^	7.1 ± 0.2^ab^	7.5 ± 0.4a
	30	6.4 ± 0.2^abc^	6.5 ± 0.3^ab^	6.1 ± 0.2^bcd^	7.0 ± 0.3a
**Texture**	1	8.6 ± 0.1^a^	8.2 ± 0.1^ab^	7.5 ± 0.3^cd^	7.8 ± 0.1bc
	30	6.8 ± 0.1^a^	6.5 ± 0.1^a^	6.5 ± 0.2^a^	6.7 ± 0.3a
**Flavor**	1	7.5 ± 0.1^cd^	7.6 ± 0.1^cd^	7.6 ± 0.2^cd^	7.4 ± 0.1d
	30	6.3 ± 0.1^bcd^	5.7 ± 0.3^e^	6.6 ± 0.1^ab^	6.9 ± 0.1a
**Overall acceptance**	1	7.7 ± 0.1^ab^	7.5 ± 0.1^ab^	7.5 ± 0.1^ab^	7.8 ± 0.1a
	30	6.5 ± 0.1^ab^	6.4 ± 0.1^abc^	6.5 ± 0.3^ab^	7.0 ± 0.3a

a Alphabetic small letters within a column showed the statistical significant difference (*P* < 0.05). The values in the tables are provided in mean ± SD.

The important point in this study was the positive effect of ajwain on the desired red color of beef sausage, so that it was able to maintain the red color of a product similar to 120 ppm sodium nitrite. Previously, the positive effect of chitosan on the color of meat products has been studied and confirmed by many researchers [67].

Regarding the sensory texture score, it was observed that at the end of the storage period, the highest score belonged to treatments control (6.4 ± 0.1), 1 % (6.8 ± 0.05), 2 % (6.5 ± 0.13), and 3 % (6.7 ± 0.28), which were significantly different (P < 0.05) from the control sample (5.5 ± 0.2). This is due to the positive effect of CEO/AEO nanoemulsion on the texture properties of mortadella samples by reducing the rate of syneresis and texture destruction due to microbial growth.

The results of sensory analysis showed that the highest flavor score was also obtained in treatments containing 1 % CEO/AEO nanoemulsion, which had a significant difference with the control sample (Table 4). As can be seen, the flavor score was highest in treatment 2 % (6.9 ± 0.1) on day 30, indicating the synergistic effect of cardamom/*C.copticum* microcapsules in preventing off-flavors in the final product. Two factors can be effective in terms of flavor, taste and odor of sausage samples. First, it is related to antimicrobial compounds, which inhibit the growth of microorganisms and reduce the proteolysis of proteins and hydrolysis of fats, and ultimately prevent off-flavor and rancidity [74]. Secondly, the presence of CEO/AEO nanoemulsion which have antioxidant and antimicrobial effects to improve the flavor, taste and odor. The use of essential oils and herbal extracts created a special herbal aroma, taste and odor in Mortadella samples, ultimately contributed to the customer-friendly product [66,75]. Finally, the panelists selected treatments control, 1, 2 and 3 % with scores of 6.5 ± 0.15, 6.4 ± 0.05, 6.5 ± 0.35 and 7.00 ± 0.32, respectively, as the best samples in terms of sensory characteristics. It should be noted that the control sample also had acceptable sensory scores (5.9 ± 0.3) and there was only a statistically significant difference with treatment 3 % (Table 4).

## 4 Conclusion

A double nanoemulsion containing *ajwain* and cardamom essential oils was prepared using the self-emulsification technique and was utilized in Mortadella sausage. GC-MS analysis of the essential oils confirmed the presence of the main compounds: thymol, ρ-cymene and γ-terpinene in the *aijwain* and 1,8-cineole, α-terpinyl acetate, and monoterpene in the cardamom. The average particle size of the nanoemulsion was less than 100 nm, demonstrating the effectiveness of self-emulsification for preparing pure or binary essential oil nanoparticles. Furthermore, the AEO and CEO nanoemulsions exhibited higher antioxidative and antibacterial activities, with a greater potency against g^+^ strains compared to g^−^ bacteria. Synergistic effects between combinations of essential oils were observed, as the nanoemulsion containing both AEO and CEO displayed enhanced antioxidant and antibacterial properties. The AEO/CEO nanoemulsion exhibited the largest growth inhibition zone for *E.coli* and *S.aureus,* along with the smallest particle size and IC_50_ values for DPPH activity. Therefore, the natural preservative system of AEO/CEO nanoemulsion was incorporated in Mortadella sausage to replace the sodium nitrite, which has carcinogenic effects. This AEO/CEO nanoemulsion demonstrated suitable textural and sensory properties in the Mortadella sausage, potentially increasing the shelf-life of beef Mortadella stored under chilled conditions. The best results were achieved with the combination of 1 % AEO/CEO nanoemulsion. Based on the findings of this study, it can be concluded that using the appropriate dose of the natural preservative can produce meat products without nitrite or, at least, reduce nitrite consumption in cooked sausage formulations to less than 50 ppm. However, further research is warranted to explore the underlying mechanisms of action regarding the appearance and nitrite reduction capabilities of the AEO/CEO nanoemulsion in meat products.

## CRediT authorship contribution statement

**Elmira Taherzadeh:** Methodology, Investigation, Formal analysis, Data curation. **Akram Arianfar:** Writing – original draft, Validation, Supervision, Software. **Elham Mahdian:** Visualization, Validation, Resources, Project administration. **Sharareh Mohseni:** Supervision, Software, Investigation, Conceptualization.

## Availability of data and materials

All the information and data will be provided based on the request after the publication. The data that support the findings of this study are not openly available and are available from the corresponding author upon reasonable request (include information on the data's location, e.g. in a controlled access repository where relevant).

## Ethics approval and consent to participate

The authors will follow the Ethical Responsibilities of Authors and COPE rules. On behalf of all co-authors, I believe the participants are giving informed consent to participate in this study.

## Consent for publication

We, Elmira Taherzadeh, Akram Arianfar, Elham Mahdian, Sharareh Mohseni give our consent for the submitted manuscript to be published in the journal of Heliyon.

## Funding

No Funding.

## Declaration of competing interest

The authors declare that they have no known competing financial interests or personal relationships that could have appeared to influence the work reported in this paper.
