# Supplementary material for: Impact of nanoemulsion of Ajwain-cardamom essential oils on Mortadella sausage quality during chilling (4°C) storage
Source: Heliyon. 2025 Jan 2;11(1):e41643. doi: 10.1016/j.heliyon.2025.e41643 (PMC11759640; doi:10.1016/j.heliyon.2025.e41643)
Supplement: Multimedia component 1 [file mmc1.docx]

Supporting Information

**Impact of nanoemulsion of Ajwain-cardamom essential oils on Mortadella sausage quality during chilling (4^o^C) storage**

*Elmira Taherzadeh ^1^, Akram Arianfar ^1*^, Elham Mahdian ^1^, Sharareh Mohseni ^2^*

*^1^ Department of Food Science and Technology, Quchan Branch, Islamic Azad University, Quchan, Iran.*

*^2^Department of Chemistry, Quchan Branch, Islamic Azad University, Quchan, Iran.*

Corresponding Author *e-mail: [a_aria_1443@yahoo.com](mailto:a_aria_1443@yahoo.com)

**Table 1.** Complete volatile profile of cardamom and ajwain identified in retention time (min) by GC-MS spectrometry.

| Flavor compound | Retention time (min) | Cardamom (%) | *Ajwain* (%) |
| --- | --- | --- | --- |
| α-Thujene | 4.998 | 0.76 | 0. 21 |
| α-Pinene | 5.098 | 1.62 | 0.23 |
| α-Phellandrene | 5.494 | - | 0.54 |
| β-Phellandrene | 5.592 | 11.01 | 0.37 |
| β-Pinene | 5.636 | 2.06 | 2.16 |
| β-Myrcene | 5.761 | 6.55 | 0.44 |
| Octanal | 5.898 | 0.24 | - |
| p-Cymene | 6.237 | - | 21.11 |
| thymol | 6.299 | - | 58.30 |
| 1,8-Cineole | 6.455 | 42.93 | 0.74 |
| β-Ocimene | 6.561 | 0.14 | 0.10 |
| γ-Terpinene | 6.774 | 0.95 | 14.72 |
| cis-Sabinenhydrate | 6.937 | 0.13 | 0.16 |
| α-Terpinolene | 7.30 | 0.30 | 1.06 |
| Linalool | 7.512 | 2.02 | - |
| Nonatriene | 7.806 | 0.13 | 0.55 |
| α-Terpineol | 9.708 | 0.53 | 1.26 |
| α-Terpinyl acetate | 11.341 | 28.62 | 0.14 |
| α-Terpinene | 14.944 | 1.97 | 0.31 |

# Gas chromatography (GC) profile of cardamom (a-c) and C. copticum (d-f)

|  |  |
| --- | --- |
|  |  |
|  |  |

**Fig. 1.** Gas chromatography (GC) profile of cardamom (a-c) and *C. copticum* (d-f).
